# HERMIONE: a randomized Phase 2 trial of MM-302 plus trastuzumab versus chemotherapy of physician’s choice plus trastuzumab in patients with previously treated, anthracycline-naïve, HER2-positive, locally advanced/metastatic breast cancer

**DOI:** 10.1186/s12885-016-2385-z

**Published:** 2016-06-03

**Authors:** Kathy Miller, Javier Cortes, Sara A. Hurvitz, Ian E. Krop, Debu Tripathy, Sunil Verma, Kaveh Riahi, Joseph G. Reynolds, Thomas J. Wickham, Istvan Molnar, Denise A. Yardley

**Affiliations:** Indiana University Melvin and Bren Simon Cancer Center, Indianapolis, IN USA; Vall d’Hebron Institute of Oncology (VHIO), Barcelona, Spain and Ramony Cajal University Hospital, Madrid, Spain; University of California Los Angeles, Los Angeles, CA USA; Dana-Farber Cancer Institute, Boston, MA USA; MD Anderson Cancer Center, Houston, TX USA; Sunnybrook Odette Cancer Centre, Toronto, Canada; Merrimack Pharmaceuticals, Inc., 1 Kendall Square, Suite B7201, Cambridge, MA 02139-1670 USA; Sarah Cannon Research Institute, and Tennessee Oncology, PLLC, Nashville, TN USA

**Keywords:** Advanced/metastatic breast cancer, Antibody–conjugate, Doxorubicin, Cardiotoxicity, HERMIONE, Human epidermal growth factor receptor 2/HER2/Erb2, HER2-targeted liposomal doxorubicin, Immunoliposome, MM­302, Trastuzumab

## Abstract

**Background:**

Human epidermal growth factor receptor 2 (HER2)-positive breast cancer is a particularly aggressive form of the disease, and ultimately progresses in patients with metastases on standard therapies. Anthracyclines, such as doxorubicin, are an effective treatment for HER2-positive breast cancer, particularly when administered in combination with trastuzumab – however, doxorubicin-related cardiotoxicity has limited its use. Many patients are therefore never treated with anthracyclines, even upon disease progression, despite the potential for benefit. MM-302 is a novel, HER2-targeted antibody–liposomal doxorubicin conjugate that specifically targets HER2­overexpressing cells. Preclinical and Phase 1 data suggest that MM-302, as a monotherapy or in combination with trastuzumab, could be effective for managing previously treated, anthracycline-naïve, HER2-positive breast cancer, without the cardiotoxicity observed with free doxorubicin formulations.

**Methods/Design:**

HERMIONE is an open-label, multicenter, randomized (1:1) Phase 2 trial of MM-302 plus trastuzumab versus chemotherapy of physician’s choice (gemcitabine, capecitabine, or vinorelbine) plus trastuzumab planned to enroll 250 anthracycline-naïve patients with locally advanced/metastatic HER2-positive breast cancer. Key inclusion criteria are: previous treatment with trastuzumab (with or without pertuzumab) in any setting; refractory or intolerant to pertuzumab (refractory to pertuzumab defined as progression in the locally advanced or metastatic setting, or disease recurrence during or within 12 months of completing pertuzumab-containing neoadjuvant and/or adjuvant therapy); and disease progression on, or intolerant to, ado-trastuzumab emtansine for locally advanced or metastatic disease. The trial is currently being conducted at sites in the USA, Canada, and Western Europe. Treatment will be administered in 21-day cycles, and will be continued until disease progression or unacceptable toxicity. The primary endpoint is independently assessed progression-free survival (PFS). Tumor response will be assessed every 6 weeks, and defined according to RECIST v1.1. Secondary endpoints include investigator-assessed PFS, overall survival (OS), OS rates at 6 months and 1 year, objective response rates, safety and tolerability, quality of life, and the pharmacokinetic profile of MM-302 plus trastuzumab.

**Discussion:**

The HERMIONE study will evaluate the efficacy and safety of MM-302 plus trastuzumab in patients with refractory HER2-positive advanced/metastatic breast cancer for whom there are no standard of care therapies with a proven survival advantage.

**Trial Registration:**

Clinicaltrials.gov identifier: NCT02213744. Registration date: 06AUG2014.

**Electronic supplementary material:**

The online version of this article (doi:10.1186/s12885-016-2385-z) contains supplementary material, which is available to authorized users.

## Background

Approximately 20 % of breast cancers are human epidermal growth factor receptor 2 (HER2)-positive, which represents a particularly aggressive subtype [1, 2]. HER2-targeted anticancer therapies such as trastuzumab, pertuzumab, ado-trastuzumab emtansine (T-DM1), and lapatinib have transformed the management and prognosis of HER2-positive advanced/metastatic breast cancer [3–10]. Current standard of care is to continue HER2-targeted therapy indefinitely (trastuzumab with changing chemotherapy partners, or T-DM1 or lapatinib in combination with capecitabine) [11–15]. Nonetheless, disease ultimately progresses in patients on standard therapies. Evidence suggests that limited mechanistic diversity associated with repeated use of microtubule-targeting chemotherapeutic agents, including taxanes (paclitaxel, nab-paclitaxel, and docetaxel) and vinca domain-binding agents (T-DM1, vinorelbine, and eribulin), could contribute to their diminishing effectiveness, owing to shared mechanisms of resistance [16]. Novel treatment strategies are essential.

### Advantages and limitations of existing anthracycline formulations

**Fig. 1:**
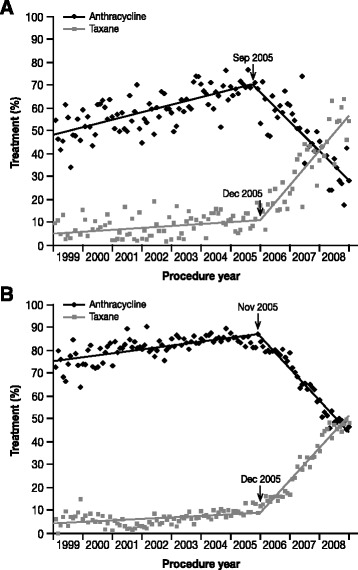
Decline in the use of anthracyclines to manage breast cancer [20]. Panels show monthly trends and fit curves of anthracycline and taxane use among **a** 4458 Medicare patients and **b** 30 422 Marketscan patients who were receiving adjuvant chemotherapy for breast cancer. Figure reproduced with permission [Copyright permission request is underway and the copyright line will be updated following manuscript acceptance]

Anthracyclines such as doxorubicin are effective for managing HER2-positive breast cancer, particularly in anthracycline-naïve patients. The trastuzumab registration study demonstrated increased efficacy with trastuzumab plus an anthracycline-containing regimen compared with an anthracycline-containing regimen alone in advanced/metastatic breast cancer [17]. However, this study also reported substantial cardiotoxicity when combining an anthracycline with trastuzumab: cardiac dysfunction occurred in 27 % of patients who received trastuzumab plus an anthracycline-based regimen (with severe heart failure reported in 16 % of patients), compared with 8 % for an anthracycline-based regimen without trastuzumab, and 13 % for paclitaxel plus trastuzumab. Furthermore, studies in the adjuvant and neoadjuvant settings suggest equivalent or greater efficacy of taxane-based regimens relative to anthracycline-based regimens in breast cancer [18, 19]. These findings have led to a decline in anthracycline use overall (Fig. 1), and particularly in the adjuvant setting in HER2-positive advanced/metastatic breast cancer [18, 20–24].

PEGylated (PLD; Doxil®/Caelyx®, Janssen) and non-PEGylated (NPLD; Myocet®, Enzon Pharmaceuticals) liposomal doxorubicin formulations have been developed that aim to overcome the challenges associated with using free doxorubicin. Both PLD and NPLD have shown reduced cardiotoxicity compared with free doxorubicin [25–32]. Although PLD appears to be active in this setting, PLD as a monotherapy or in combination with trastuzumab is not considered a standard of care in the treatment of HER2-positive advanced/metastatic breast cancer. In Europe, PLD monotherapy is approved for metastatic breast cancer patients at high cardiac risk [33], but in the USA, PLD has not been approved either alone or in combination with trastuzumab. NPLD in combination with trastuzumab and paclitaxel was not superior to the combination of trastuzumab and paclitaxel in a Phase 3 study of first-line therapy for advanced/metastatic breast cancer [32].

### MM-302 – a novel, HER2-targeted antibody*–*liposomal doxorubicin conjugate

**Fig. 2:**
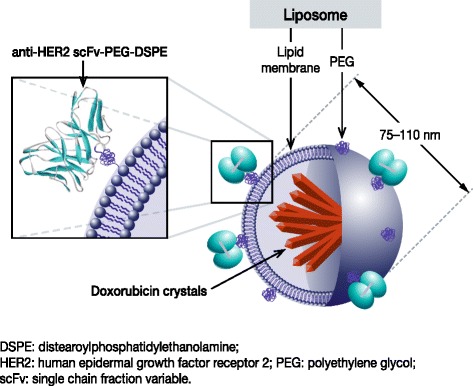
Schematic of MM-302, a novel HER2-targeted antibody–liposomal doxorubicin conjugate. MM-302 consists of doxorubicin encapsulated by a liposome that is conjugated to an anti-HER2 scFv antibody via a polyethylene glycol spacer (PEG-DSPE). MM-302 thus directly targets PEGylated liposomal doxorubicin to HER2-overexpressing tumor cells

**Fig. 3:**
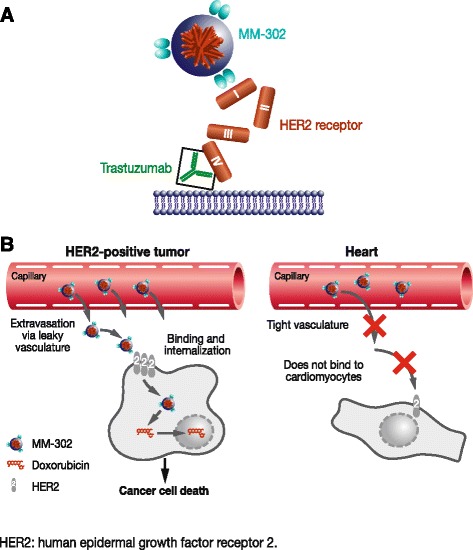
Mechanism of action of MM-302. **a** MM-302 binds to HER2 extracellular subdomain I, whilst trastuzumab binds to subdomain IV. **b** MM-302 remains in circulation for long periods of time, providing an opportunity to accumulate in tumors via leaky vasculature. Once in the tumor microenvironment, MM-302 binds specifically to tumor cells that overexpress HER2 (>200 000/cell) and undergoes receptor-mediated endocytosis, releasing doxorubicin inside the cell. By contrast, the vasculature of the heart is more intact and prevents extravasation out of the blood vessels. Furthermore, cardiomyocytes express HER2 below the threshold required for uptake; therefore, MM-302 does not inhibit HER2-mediated signaling in cardiomyocytes [34, 35]

MM-302 is a novel, HER2-targeted antibody–liposomal doxorubicin conjugate (Fig. 2) that specifically targets HER2­overexpressing cells, increasing delivery of doxorubicin to tumor cells and limiting exposure to healthy cells such as cardiomyocytes (Fig. 3). MM-302 is therefore designed potentially to be better tolerated than free doxorubicin, and to be more effective than free doxorubicin and existing liposomal formulations in HER2-overexpressing tumors [34, 35]. In preclinical models, MM-302 demonstrated superior antitumor activity compared with both free doxorubicin and PLD [35]. Moreover, MM­302 and trastuzumab bind to different HER2 epitopes, and the combination of MM-302 and trastuzumab demonstrated superior antitumor activity to either agent alone in HER2­overexpressing tumor xenograft models (unpublished observations, Chris Espelin, Shannon Leonard, and Elena Geretti; Merrimack Pharmaceuticals, Inc., Cambridge, MA, USA). These findings provided support for investigating the combination in the clinical setting. Phase 1 data suggest that MM-302 alone or in combination with trastuzumab has promising efficacy and a manageable safety profile in patients with advanced HER2-positive breast cancer. In the population treated with ≥30 mg/m^2^ MM-302 alone (*n* = 34) or combined with either trastuzumab (*n* = 22) or trastuzumab plus cyclophosphamide (*n* = 13), the objective response rate was 11.3 % (*n* = 7/62) and median progression-free survival (PFS) was 7.6 months (95 % CI: 3.6–10.9). Increased efficacy was observed in the anthracycline-naïve subgroup, with an objective response rate of 24.0 % (*n* = 6/25) and median PFS of 11.0 months (95 % CI: 1.8–13.1) [36]. No cardiac adverse events were reported with MM-302 monotherapy; in the combination arms, cardiac adverse events were infrequent and none were serious adverse events [36].

### Rationale for the HERMIONE trial

Given the high unmet need for novel treatment options for patients with HER2- positive metastatic breast cancer that has progressed on pertuzumab, trastuzumab, and T-DM1, and the promising activity seen in the Phase 1 study, the Phase 2 HERMIONE trial has been designed, with input from the US Food and Drug Administration (FDA) and review by the European Medicines Agency (EMA), to assess the efficacy and tolerability of MM-302 in combination with trastuzumab, compared with chemotherapy of physician's choice plus trastuzumab, in patients with previously treated, anthracycline-naïve, HER2­positive locally advanced/metastatic breast cancer.

## Methods/Design

### Study design

**Fig. 4:**
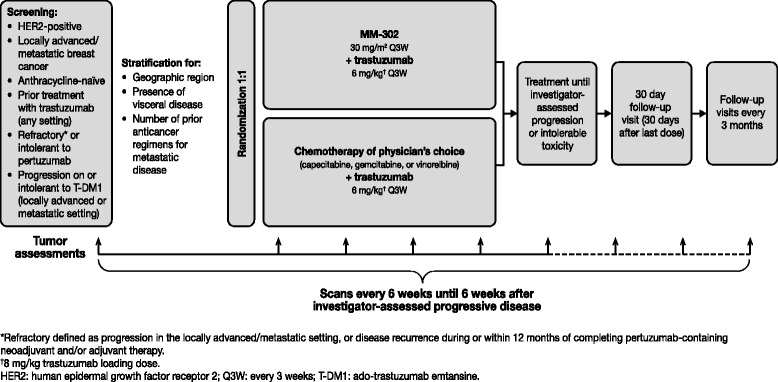
HERMIONE study design and assessment schedule

HERMIONE is an open-label, multicenter, randomized (1:1) Phase 2 trial of MM-302 plus trastuzumab versus chemotherapy of physician’s choice (gemcitabine, capecitabine, or vinorelbine) plus trastuzumab in anthracycline-naïve patients with locally advanced/metastatic HER2-positive breast cancer (ClinicalTrials.gov Identifier: NCT02213744). The trial is being conducted at sites in the USA, Canada, and Western Europe. The trial design and treatment schedule are summarized in Fig. 4.

Patients will be randomized according to a pre-specified randomization scheme generated by an independent statistician. Once patient eligibility for the study has been confirmed, investigational sites will log into a computerized interactive web response system to obtain treatment arm assignment. Randomization will be stratified based on world region (North America/Western Europe/Other), presence of visceral disease (yes/no), and number of prior lines of anticancer therapy for locally advanced/metastatic disease (≤3/≥4).

The study will be performed according to the principles of the Declaration of Helsinki, the International Conference on Harmonization Guidance on Good Clinical Practice, and the requirements of the US FDA and/or local regulatory authorities regarding the conduct of human clinical trials. All patients must give written informed consent. Local Institutional Review Board or Ethics Committee approval of the protocol, informed consent document, and any other material used to inform the patient about the nature of the trial has been obtained for all participating centers.

### Eligibility criteria

**Fig. 5:**
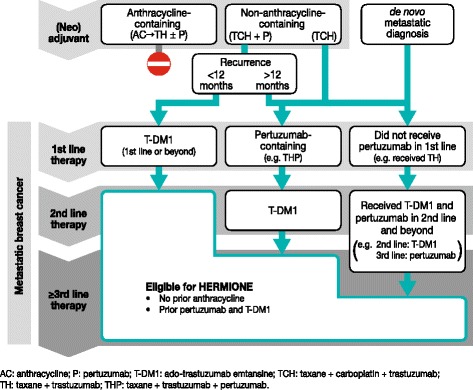
Examples of the most common previous treatment pathways for eligible patients

Key inclusion criteria are: histologically or cytologically confirmed invasive breast cancer that is centrally confirmed as HER2-positive by American Society of Clinical Oncology/College of American Pathologists 2013 guidelines [37]; documented locally advanced/metastatic disease; prior treatment with trastuzumab in any setting; refractory or intolerant to pertuzumab (refractory to pertuzumab is defined as progression on pertuzumab in the locally advanced or metastatic setting, or development of disease recurrence during or within 12 months of completing pertuzumab-containing neoadjuvant and/or adjuvant therapy); disease progression on, or intolerant to, T-DM1 treatment for locally advanced or metastatic disease; not previously treated with an anthracycline in any setting; aged ≥18 years; Eastern Cooperative Oncology Group performance status 0 or 1; left ventricular ejection fraction (LVEF) ≥50 %. Patients with treated, stable, asymptomatic central nervous system (CNS) metastases and who have not received steroid treatment for 4 weeks prior to enrollment may be considered for this trial. Further details of the study inclusion and exclusion criteria are presented in Table 1, and examples of the most common previous treatment pathways for eligible patients are shown in Fig. 5. There is no restriction on the number of prior lines of therapy for eligible patients.

**Table 1:** Key inclusion and exclusion criteria for the Phase 2 HERMIONE study

Criteria	Details
Inclusion criteria	
Disease-specific	• Histologically or cytologically confirmed invasive cancer of the breast, with documented locally advanced/metastatic disease that is not amenable to resection with curative intent. Cancer must be HER2-positive, as defined by ASCO/CAP 2013 guidelines [37]. • Documented disease progression (via RECIST or clinical progression) or intolerance during or after the most recent treatment for locally advanced/metastatic breast cancer. • Refractory or intolerant to pertuzumab (refractory to pertuzumab is defined as progression on pertuzumab in the locally advanced or metastatic setting, or development of disease recurrence during or within 12 months of completing pertuzumab-containing neoadjuvant and/or adjuvant therapy). • Disease progression on, or intolerant to, T-DM1 in the locally advanced/metastatic breast cancer setting. • Previously treated with trastuzumab in any setting (trastuzumab could have been previously administered with or without pertuzumab).
General	• Age ≥18 years. • Eastern Cooperative Oncology Group performance status 0 or 1.
Hematologic, biochemical, and organ function	• Eligible to receive at least one of gemcitabine, capecitabine, or vinorelbine. • Adequate bone marrow reserves (absolute neutrophil count ≥1500/μL; platelet count ≥100 000/μL; hemoglobin ≥9 g/dL [transfusions allowed]), coagulation function (INR and aPTT ≤1.5 ULN, unless on therapeutic coagulants), hepatic function (serum total bilirubin within normal limits; AST and ALT up to 3x ULN; serum albumin ≥2.5 g/dL), renal function (serum creatinine ≤1.5x ULN), and cardiac function (LVEF ≥50 % by MUGA or ECHO).
Exclusion criteria	
Disease-specific	• Previous treatment with doxorubicin, liposomal doxorubicin, epirubicin, mitoxantrone, or any other anthracycline derivative. • CNS metastases, unless patients have been treated and are stable without symptoms for 4 weeks after completion of treatment, and they must be off steroids for at least 4 weeks prior to enrollment. • Active other malignancy or history of other malignancy within the last 5 years except appropriately treated carcinoma of the cervix, non-melanoma skin carcinoma, stage 1 uterine cancer, or cancers with a similar curative outcome as those previously mentioned. • Known hypersensitivity to any of the components of MM-302 or hypersensitivity reactions to fully humanized monoclonal antibodies. • History of intolerance to trastuzumab. Patients who have been successfully re-challenged with trastuzumab after a mild infusion reaction are allowed. • Investigational therapy administered <28 days (or <5 half-lives; whichever is the longest) prior to the first scheduled day of study drug dosing. • Any standard anticancer therapy <14 days prior to the first scheduled day of study drug dosing (except trastuzumab).
Cardiac	• Any class of NYHA congestive heart failure, or heart failure with preserved ejection fraction. • History of known coronary artery disease or a myocardial infarction within the last 12 months. • Uncontrolled hypertension (SBP >160 mmHg or DBP >100 mmHg). • Unstable angina pectoris. • Known history of serious cardiac arrhythmias requiring treatment (exception: atrial fibrillation and paroxysmal supraventricular tachycardia). • Prolonged QTc interval (≥450 ms). • Previous discontinuation of trastuzumab due to unacceptable cardiac toxicity or infusion-related reactions. • History of LVEF decline to <50 % during or after HER2-directed therapy. • Current dyspnea at rest that requires continuous oxygen therapy.
General	• Pregnant or breast feeding. • Active infection or unexplained fever >38.5 °C during screening visits. • History of allogeneic transplant (patients with a history of autologous bone marrow or stem cell transplant may be enrolled).

*ALT* alanine aminotransferase, *aPTT* activated partial thromboplastin time, *ASCO* American Society of Clinical Oncology, *AST* aspartate aminotransferase, *CAP* College of American Pathologists, *CNS* central nervous system, *CTCAE* Common Terminology Criteria for Adverse Events, *DBP* diastolic blood pressure, *ECHO* echocardiogram, *HER2* human epidermal growth factor receptor 2, *INR* international normalized ratio, *LVEF* left ventricular ejection fraction, *MUGA* multiple-gated acquisition scan, *NYHA* New York Heart Association, *QTc* corrected QT interval, *RECIST* Response Evaluation Criteria In Solid Tumors, *SBP* systolic blood pressure, *T-DM1* ado-trastuzumab emtansine, *ULN* upper limit of normal

### Study treatments

#### Treatment arms

Patients will be randomized to receive either MM-302 plus trastuzumab or chemotherapy of physician's choice plus trastuzumab. In the experimental arm, patients will receive MM-302 30 mg/m^2^ IV on day 1 of each 21-day cycle, and trastuzumab 8 mg/kg IV (loading dose) and 6 mg/kg IV (maintenance dose) on day 1 of each 21-day cycle. In the control arm, physicians will select a chemotherapy (limited to gemcitabine, capecitabine, or vinorelbine) plus trastuzumab, as follows: gemcitabine 1000–1250 mg/m^2^ IV on days 1 and 8 of each 21-day cycle; capecitabine 1000–1250 mg/m^2^ twice daily, administered orally on days 1–14 of each 21-day cycle; vinorelbine 25–30 mg/m^2^ IV on days 1 and 8 (and optionally on day 15) of each 21-day cycle; trastuzumab administration in the control arm is the same as in the experimental arm. Treatment will be continued until progression or intolerable toxicity. There will be no crossover of control arm to receive study drug on progression.

#### Dose modifications

Dose modification of study treatments is permitted to manage toxicities. A maximum of two MM-302 dose reductions (by 25 %) are permitted to manage hematologic and non-hematologic adverse events. For hepatotoxicity, the dose will be reduced to 15 mg/m^2^ if total bilirubin is 1.2–3.0 mg/dL, and to 7.5 mg/m^2^ if total bilirubin is >3.0 mg/dL. Any patients requiring a third dose reduction will have MM-302 discontinued. Specific MM-302 dose modification criteria are also defined for managing changes in LVEF. In case of persistent asymptomatic LVEF decreases and congestive heart failure, study treatment will be permanently discontinued. Patients with confirmed symptoms of congestive heart failure will also discontinue treatment permanently.

Specific criteria to withhold/discontinue MM-302 treatment are also defined for managing LVEF changes. MM-302 will be withheld if LVEF declines to ≤45 % or if LVEF declines to 46–49 % and is ≥15 % points below baseline. LVEF assessment will then be repeated after 3 weeks: if LVEF recovers sufficiently (LVEF ≥50 %, or 46–49 % and <15 % points below baseline), study treatment will be resumed, otherwise the patient will permanently discontinue treatment.

#### Concomitant therapies

Patients may receive concomitant analgesics, antiemetics, antidiarrheal antibiotics, antipyretics, hematopoietic growth factors, and blood products as per physician discretion. For patients with bone metastases, standard of care treatments such as bisphosphonates and denosumab are permitted. The following therapies are not permitted while on study treatment: other antineoplastic therapies; radiotherapy (unless given for palliative reasons and with the approval of the Medical Monitor); systemic prophylactic corticosteroids prior to first administration of MM-302; any other investigational therapies; and any therapies that are prohibited from being given concomitantly with gemcitabine, capecitabine, or vinorelbine, as described in the respective national prescribing information.

### Study objectives

The primary objective is to determine whether the combination of MM-302 plus trastuzumab improves PFS (time from randomization until documented disease progression or death) compared with chemotherapy of physician's choice plus trastuzumab. PFS will be assessed by a blinded independent review. Secondary endpoints are investigator-assessed PFS; overall survival (OS; time from randomization until death); OS rate at 6 months and 1 year; time to treatment failure (time from randomization until treatment discontinuation for any reason, including disease progression, treatment toxicity, or death); objective response rate (patients with confirmed complete or partial response); duration of response (time from first, documented, confirmed objective response until objectively documented recurrent or progressive disease); safety profiles; and pharmacokinetic exposure of MM-302 and trastuzumab. Exploratory objectives include the correlation between biomarkers and clinical outcome; time to symptomatic progression; patient-reported outcomes (Functional Assessment of Cancer Therapy-Breast [FACT-B] and EuroQol [EQ]-5D); population pharmacokinetics of MM-302 including estimations of inter-patient variability of pharmacokinetic parameters; rate and time to development of CNS progression and new CNS metastases; estimation of resources for hospitalizations/hospital visits during the study and within 30 days of the last dose of study treatment; and concordance between independently and investigator-assessed PFS. An independent Cardiac Review Committee will monitor cardiac safety, and a Data Safety Monitoring Board will monitor the overall safety of the study.

### Study assessments

Tumor response will be assessed by computed tomography/magnetic resonance imaging at screening and every 6 weeks from randomization, and will be defined according to Response Evaluation Criteria In Solid Tumors (RECIST) v1.1. Cardiotoxicity will be assessed by monitoring LVEF at screening and every 6 weeks from randomization until 6 weeks after treatment discontinuation. LVEF will be determined by multiple-gated acquisition scan or echocardiogram, with the same assessment method continued throughout the study. Long-term cardiotoxicity will be assessed by recording the occurrence of symptomatic heart failure in the follow-up period of the study. Safety will be assessed using the National Cancer Institute Common Terminology Criteria for Adverse Events (NCI-CTCAE) v4.0 at screening; on days 1, 8, and 15 of cycle 1; on day 1 of every cycle thereafter; and 30 days after treatment discontinuation. Blood samples for pharmacokinetic analyses will be taken in cycles 1 and 2 from patients randomized to MM­302 and trastuzumab; samples will be collected pre-infusion, immediately post-infusion, 8–96 h post-infusion (optional sample), and 168 h post-infusion. Patient quality of life will be assessed using the FACT-B and EQ-5D instruments. For analysis of serum biomarkers (in patients who consent) and anti-MM-302 immunogenicity (in the MM-302 arm only), serum blood samples will be collected before dosing on day 1 of cycle 1 and every cycle thereafter and 30 days after treatment discontinuation.

### Statistical analysis

The planned sample size is approximately 250 patients (125 per arm). This sample size was chosen to observe 191 events, providing at least 90 % power to detect an approximately 60 % improvement in PFS (hazard ratio of 0.625). The study powering assumes a median PFS of 8 months and 5 months in the experimental arm and control arm, respectively.

Efficacy will be assessed in the intent-to-treat population, which will include all randomized patients. Time-to-event analyses, including the primary endpoint, will be compared between the experimental and control arms using a stratified log-rank test at a 2­sided 5 % significance level in the intent-to-treat population. Time-to-event curves will be estimated using Kaplan–Meier estimates. Hazard ratios and 95 % confidence intervals will be estimated using Cox proportional hazard models. Objective response rate will be assessed using a stratified Mantel–Haenszel test. The safety population will include all patients who receive at least one dose (including a partial dose) of study medication; safety will be assessed using descriptive statistics.

## Discussion

The Phase 2 HERMIONE trial has been designed, with input from the US FDA and review by the EMA, to assess the efficacy and tolerability of MM-302 in combination with trastuzumab, compared with chemotherapy of physician's choice plus trastuzumab, in anthracycline-naïve patients with HER2­positive locally advanced/metastatic breast cancer previously treated with pertuzumab or T-DM1. Recruitment to the trial began in September 2014.

HERMIONE is investigating a patient population with a high unmet need for effective therapies. The standard of care for advanced/metastatic HER2-positive breast cancer is to continue HER2-targeted therapy indefinitely (trastuzumab with changing chemotherapy partners, or T-DM1 or lapatinib in combination with capecitabine) [11–15]. Microtubule-targeting chemotherapies, including taxanes (paclitaxel, nab-paclitaxel, and docetaxel) and vinca domain-binding agents (T-DM1, vinorelbine, and eribulin), are frequently used. However, common resistance mechanisms are likely to occur among microtubule-targeted agents and especially within the specific classes [16]. These shared resistance mechanisms could result in diminishing treatment benefit for patients. In addition, successive use of microtubule-targeting agents may pose increasing safety concerns due to cumulative neuropathy. Apart from the microtubule-targeting agents, the most frequently used chemotherapeutics in combination with trastuzumab include capecitabine, and gemcitabine [8, 15]. Many patients continue to have a good performance status after disease progression on standard HER2-targeted therapies and continue to be candidates for additional treatment. Given the heterogeneity of advanced breast cancer, a mechanistically diverse range of therapeutic options is warranted.

The study was designed to recruit anthracycline-naïve patients following preliminary efficacy data suggesting that treatment with MM-302 results in higher response rates and longer median PFS in anthracycline-naïve patients, compared with those previously exposed to anthracyclines [36, 38], consistent with published literature on PLD [26, 39, 40]. In addition, the safety concerns over the use of anthracyclines in combination with trastuzumab have led to a decline in anthracycline use, resulting in a large pool of patients who are anthracycline-naïve, and who have not received one of the most active classes of chemotherapy for the treatment of breast cancer [20]. The development of novel formulations of these agents may therefore extend the benefits of this class to this group of patients.

Patients enrolling in HERMIONE who have already been exposed to trastuzumab will continue trastuzumab treatment. As noted earlier, this is in line with the current standard of care for HER2-positive breast cancer of continuing HER2-targeted therapy indefinitely [11–15]. Supporting this practice, a separate clinical study randomized patients with HER2-positive breast cancer whose disease had progressed on trastuzumab treatment to receive capecitabine alone or capecitabine plus trastuzumab. The study results showed a significant improvement in overall response and PFS with capecitabine plus trastuzumab compared with capecitabine alone [12, 13], supporting the continued use of trastuzumab plus chemotherapy in HER2-positive breast cancer.

The MM-302 dose of 30 mg/m^2^ every 21 days was selected based on safety and efficacy data from an ongoing Phase 1 trial: MM-302 was well tolerated at doses of 30–40 mg/m^2^ but an increase in grade 3/4 adverse events was observed at 50 mg/m^2^ administered every 4 weeks (the highest dose level evaluated) [36, 38]. The 21-day cycle for MM-302 was selected to simplify combination with the approved trastuzumab regimen, as this is also administered every 21 days [38].

The control arm will receive trastuzumab added to chemotherapy of physician's choice, either gemcitabine, capecitabine, or vinorelbine. These are three of the most commonly used cytotoxic agents (besides taxanes, which will have been used in earlier lines of therapy); they can be combined with trastuzumab [8, 12, 13, 41–44]; and they are recommended in treatment guidelines [11, 14, 15]. Chemotherapy of physician's choice is limited to only three agents to minimize potential confounding of the key outcome measures, given the relatively small sample size required for this study. There is flexibility in the dose ranges that physicians can use, in line with the approved US package insert or European Summary of Product Characteristics, and the National Comprehensive Cancer network guidelines [15, 45–50], reflecting doses commonly used in the clinic.

The primary endpoint for the HERMIONE trial is PFS as determined by a blinded independent review, based on evidence that increases in PFS have generally translated into improvements in OS in late-line HER2-positive advanced/metastatic breast cancer. An analysis of approval trials in HER2-positive advanced/metastatic breast cancer suggested a moderate correlation between PFS and OS [51]. Furthermore, the TH3RESA trial reported a PFS hazard ratio of 0.53 that corresponded with an OS hazard ratio of 0.55 from the first interim analysis [8]. This trial evaluated T-DM1 in HER2-positive late-line advanced/metastatic breast cancer, and used a comparator arm of treatment of physician’s choice, which was predominantly trastuzumab in combination with chemotherapy. These data suggest that an improvement in PFS with trastuzumab in combination with MM-302 in a similar patient population to that in TH3RESA could likewise translate into an improvement in OS.

## Conclusion

The HERMIONE trial is designed to test whether MM-302, a novel HER2-targeted antibody–liposomal doxorubicin conjugate, added to trastuzumab could be an effective and well­tolerated treatment option for anthracycline-naïve patients with HER2-positive advanced/metastatic breast cancer who are candidates for systemic therapy after disease progression on approved agents.

## Abbreviations

CNS, central nervous system; EMA, European Medicines Agency; EQ, EuroQol; FACT-B, functional assessment of cancer therapy-breast; FDA, Federal Drug Administration; HER2, human epidermal growth factor receptor 2; LVEF, left ventricular ejection fraction; NCI-CTCAE, National Cancer Institute Common Terminology Criteria for Adverse Events; NPLD, non-PEGylated liposomal doxorubicin; OS, overall survival; PFS, progression-free survival; PLD, PEGylated liposomal doxorubicin; RECIST, response evaluation criteria in solid tumors; T-DM1, Ado-trastuzumab emtansine; VHIO, Vall d’Hebron Institute of Oncology.

## Additional file

Additional file 1: HERMIONE IRB Data. (XLSX 15 kb)
